# Comprehensive analysis of LMNB2 in pan-cancer and identification of its biological role in sarcoma

**DOI:** 10.18632/aging.205962

**Published:** 2024-07-08

**Authors:** Yonghui Guo, Min Zhang, Yingrui Luo, Yingshi Li, Yanxia Xu, Nisha Wang

**Affiliations:** 1 Department of Laboratory Medicine, Zhujiang Hospital, Southern Medical University, Guangzhou, Guangdong Province, China; 2 Guangdong Provincial Clinical Research Center for Laboratory Medicine, Guangzhou, Guangdong Province, China; 3 GuangDong Second Province General Hospital, Neurosurgery Department (MH), Guangzhou, Guangdong Province, China; 4 Cancer Research Institute, School of Basic Medical Sciences, Southern Medical University, Guangzhou, Guangdong Province, China; 5 Southern Medical University, Guangzhou, Guangdong Province, China; 6 Department of Radiation Oncology, Zhuhai People’s Hospital (Zhuhai Clinical Medical College of Jinan University), Zhuhai, Guangdong Province, China; 7 Department of Biochemistry and Molecular Biology, School of Basic Medical Sciences, Southern Medical University, Guangzhou, Guangdong Province, China

**Keywords:** LMNB2, pan-cancer, sarcoma, prognosis, cell proliferation

## Abstract

Backgrounds: Sarcoma (SARC) is a mesenchymal tumor which often responds poorly to systemic therapy. It is therefore important to look for possible biological markers that could tell the prognosis and the progression of SARC.

Methods: A combined evaluation of the Cancer Genome Atlas (TCGA) and genotypic tissue expression (GTEx) portal was used to analyzeLMNB2 expression level in different types of cancer. Kaplan-Meier survival analysis was performed to examine LMNB2 predictive value in over-all survival rate and disease-free survival rate. The association among LMNB2 expression level and immune cell infiltration, microsatellite instability (MSI) and tumor mutational burden (TMB) were analyzed. GO and KEGG enrichment analysis were performed to predicate LMNB2 biological functions. The biological function of LMNB2 was estimated by MTT and flow cytometry assay. Additionally, western blot assay was used to examine protein expression levels.

Results: Increased LMNB2 expression was related with worsened cancer type-dependent survival. A relation between LMNB2 expression levels and immune cell infiltration was found. GO and KEGG enrichment analysis indicated that LMNB2 was involved in a series of pathways. Biology function assays revealed that down-regulation of LMNB2 impaired proliferation and cell cycle distribution. At the mechanical level, LMNB2 acts as a regulator of cyclinD1 and cyclinE1.

Conclusions: Altogether, these data suggest that LMNB2 may serve as a tumor promoter and could be a possible target for cancer therapy.

## INTRODUCTION

Sarcomas (SARC) form a heterogeneous cancer population from mesenchymal cell sources consisting of more than 70 malignant primary tumors [1]. The most common sarcomas start in the bone and soft tissue and are responsible for approximately 19 to 21% of cancer-related deaths in children and young adults [2, 3]. Standard treatment options include neoadjuvant chemotherapy combined with surgery and/or radiotherapy [4]. However, new treatments are needed because the overall survival rate of patients with metastatic or recurrent diseases is less than 20% [5]. In the last two decades, the understanding of the molecular pathogenesis of sarcoma has increased, but this has not been translated into effective diagnostic and prognostic methods.

The nuclear lamina mainly contains 4 proteins-lamin A, which transcribed from LMNA encoding lamin A and lamin C, and lamin B1 (LMNB1) and lamin B2 (LMNB2). The human Lamin B2 (LMNB2) gene encodes a 68 kDA protein and is localized on chromosome 19p13.3. LMNB2 takes part in chromatin structure, gene expression and nuclear stability [6]. Overexpression of LMNB2 has been found in a range of cancers and abnormal expression of LMNB2 often contributes to cancer progression [7]. However, the role of LMBB2 in SARC is still poorly understood. We thus chose LMBB2 for further study.

This study reports on LMNB2 expression in pan-cancer and particularly on the effect it has on SARC cell proliferation. Furthermore, the molecular mechanism of LMNB2 in SARC cells is explained. Better understanding is gained on SARC pathogenesis and the possible significance of LMNB2 as a therapeutic target for sarcoma.

## RESULTS

### Comprehensive analysis of LMNB2 in pan-cancers

**Figure 1:**
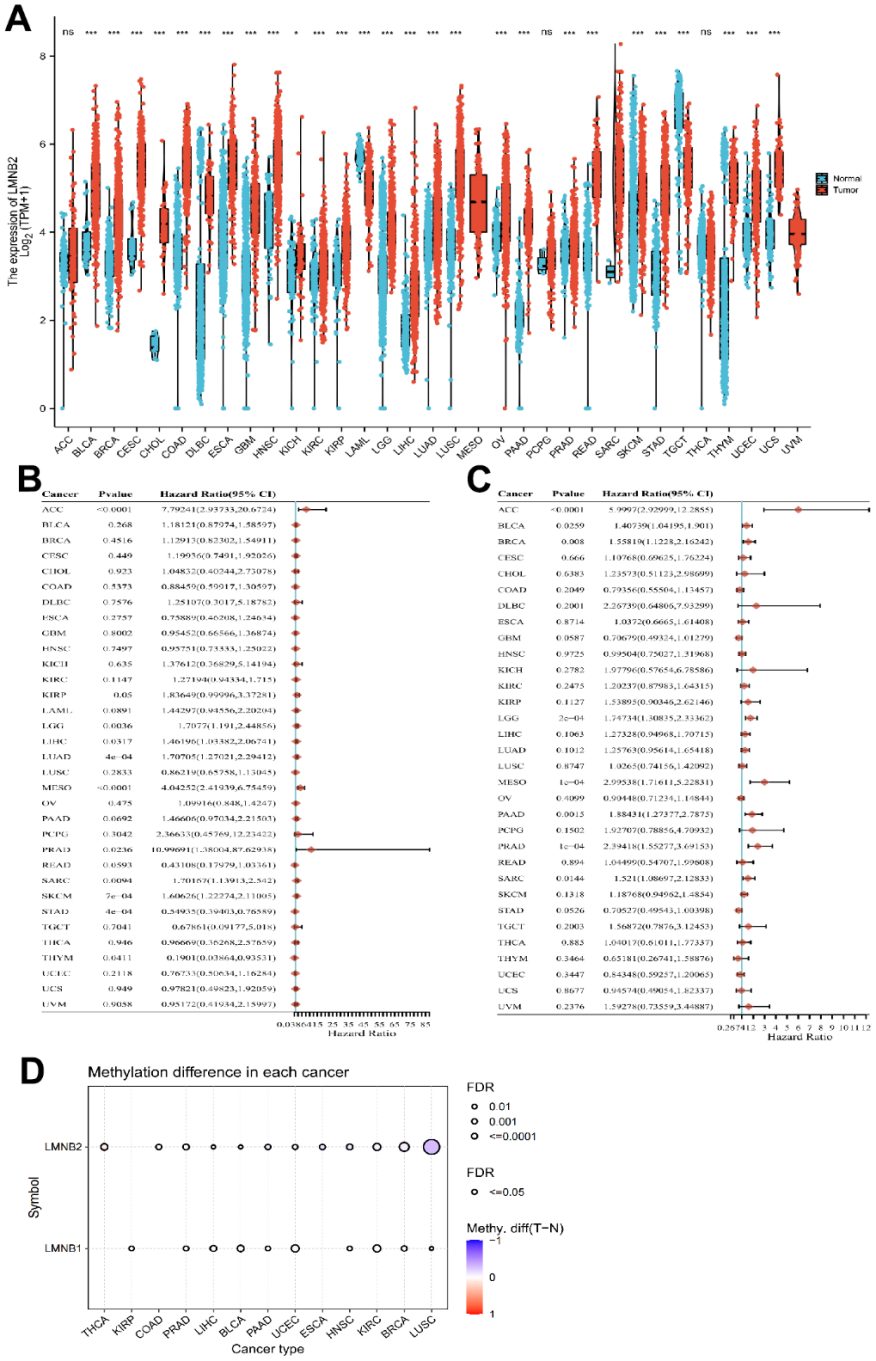
**Comprehensive analysis of LMNB2 in pan-cancers.** (**A**) LMNB2 expression levels in different cancers from the TCGA database were demonstrated (** P<.01; *** P<.001). (**B**) Forest plot shows the univariate Cox regression analysis results for LMNB2 to predict the OS in pan-cancers from the TCGA database. (**C**) A Forest plot shows the univariate Cox regression analysis results for LMNB2 to predict PFS in pan-cancers from the TCGA database. (**D**) The methylation status of LMNB2 in pan-cancer was demonstrated.

First, we analyzed the expression of LMNB2 indifferent cancer types by mining the TCGA database. When para-cancerous tissue was compared with non-cancer tissue, LMNB2 mRNA showed high expression levels in all types of cancers, expect in adenoid cystic carcinoma (ACC), pheochromocytoma and paraganglioma (PCPG) and thyroid carcinoma (THCA). In acute myeloid leukemia (LAML), LMNB2 mRNA was down-regulated in cancer tissues when compared with normal tissues. In mesothelioma (MESO) and uveal melanoma (UVM), the TCGA database lacked the paracancerous and normal tissues data for analysis (Figure 1A). The association between the LMNB2expression levels and OS rate across the 33 different cancers from the TCGA database was also analyzed. The forest plots revealed that an up-regulated LMNB2 expression level was associated with poor OS in ACC, kidney renal papillary cell carcinoma (KIRP), brain lower grade glioma (LGG), liver hepatocellular carcinoma (LIHC), lung adenocarcinoma (LUAD), MESO, prostate adenocarcinoma (PRAD), SARC and skin cutaneous melanoma (SKCM). However, in thymoma (THYM) and stomach adenocarcinoma (STAD), the up-regulation of LMNB2 was related to a better OS, which meant LMNB2 seemed to have a more protective role in these two cancers (Figure 1B). In addition, we revealed that up-regulated expression levels of LMNB2 were associated with shorter PFS in ACC, bladder urothelial carcinoma (BLCA), breast invasive carcinoma (BRCA), LGG, MESO, pancreatic adenocarcinoma (PAAD), PRAD and SARC (Figure 1C). Epigenetic modification plays an important role in mRNA expression level, we thus examined the methylation status of LMNB2 in pan-cancer. As revealed in Figure 1D, LMNB2 methylation was often found in BRCA and LUSC.

The association between LMNB2 expression levels and molecular subtypes across cancers was analyzed by using the TISIDB database (http://cis.hku.hk/TISIDB). LMNB2 expression was significantly associated with the molecular subtype of ACC, BRCA, colon adenocarcinoma (COAD), esophageal carcinoma (ESCA), glioblastoma multiforme (GBM), KIRP, head and neck squamous cell carcinoma (HNSC), LGG, lung squamous cell carcinoma (LUSC), LIHC, ovarian Serous cystadenocarcinoma (OV), PCPG, STAD and uterine corpus endometrial carcinoma (UCEC) (Supplementary Figure 1). Taken together, the above findings uncover that LMNB2 may be an oncogene in most cancers.

The TISIDB database was also used to check the correlation of LMNB2 expression with lymphocytes, immunomodulators and chemokines in pan-cancer (Supplementary Figure 2). The results demonstrated that LMNB2 may modulate the immune response across cancers.

The relation between LMNB2 expression and tumor mutational burden (TMB) was analyzed. The expression of LMNB2 was positively related with TMB in LGG, BRCA, LUAD, STAD, UCEC, ACC, PRAD, SARC, SKCM, rectum adenocarcinoma (READ), PAAD, BLCA, uterine carcinosarcoma (UCS), kidney chromophobe (KICH), cholangiocarcinoma (CHOL), LUSC and MESO, but negatively related with TMB in THYM (Supplementary Figure 3A). LMNB2 expression and microsatellite instability (MSI) association was also analyzed. LMNB2 expression was positive associated with MSI in LUSC, UCEC, STAD, UCS, LUAD, PRAD, kidney renal clear cell carcinoma (KIRC), KICH, testicular germ cell tumors (TGCT) and SARC, but negatively associated with MSI in READ and lymphoid neoplasm diffuse large B-cell lymphoma (DLBC) (Supplementary Figure 3B).

Finally, the association among LMNB2 expression levels and different immune markers across cancers was analyzed. It was found that LMNB2 expression levels were significantly negatively associated with these markers in TGCT, but positively associated with the markers in SARC (Supplementary Figure 3C).

### LMNB2 co-expression network and enrichment pathway analysis in pan-cancer

**Figure 2:**
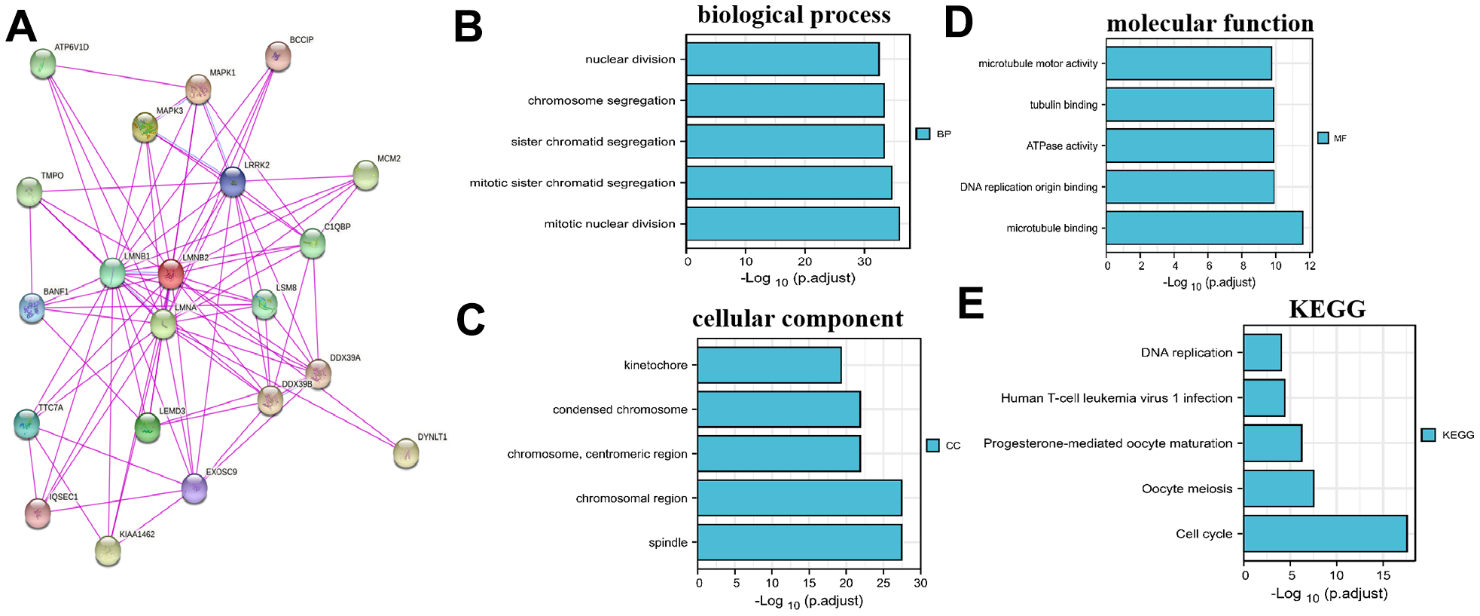
**LMNB2Co-expression network and enrichment pathway analysis.** (**A**) Protein-protein interactions of LMNB2 from the STRING website. (**B**–**E**) Biological processes, cellular components, molecular functions and KEGG pathways for LMNB2 binding proteins and interactive genes.

The binding proteins of LMNB2 were predicted by the STRING website and validated. The result revealed that there were 19 proteins binding to LMNB2 (ATP6V1D, TMPO, BANF1, TTC7A, IQSEC1, LMNB1, KIAA1462, MAPK3, LMNA, LEMD3, EXOSC9, MAPK1, LRRK2, LSM8, DDX39B, C1QBP, DDX39A, MCM2, DYNLT1, Figure 2A). We obtained the top 100 genes that were closest related to LMNB2 from the gene expression profiling interactive analysis database (GEPIA, http://gepia2.cancer-pku.cn/). GO enrichment analysis of the 119 genes set showed that they were significantly related with sister chromatid segregation, chromosome segregation, mitotic sister chromatid segregation, mitotic nuclear division, the kinetochore, DNA replication origin binding, ATPase activity and other functions (Figure 2B–2D). The KEGG enrichment analysis showed LMNB2 to be engaged in Human T-cell leukemia virus 1 infection, DNA replication, oocyte meiosis, progesterone-mediated oocyte maturation and the cell cycle (Figure 2E).

### LMNB2 expression is elevated in SARC patients

**Figure 3:**
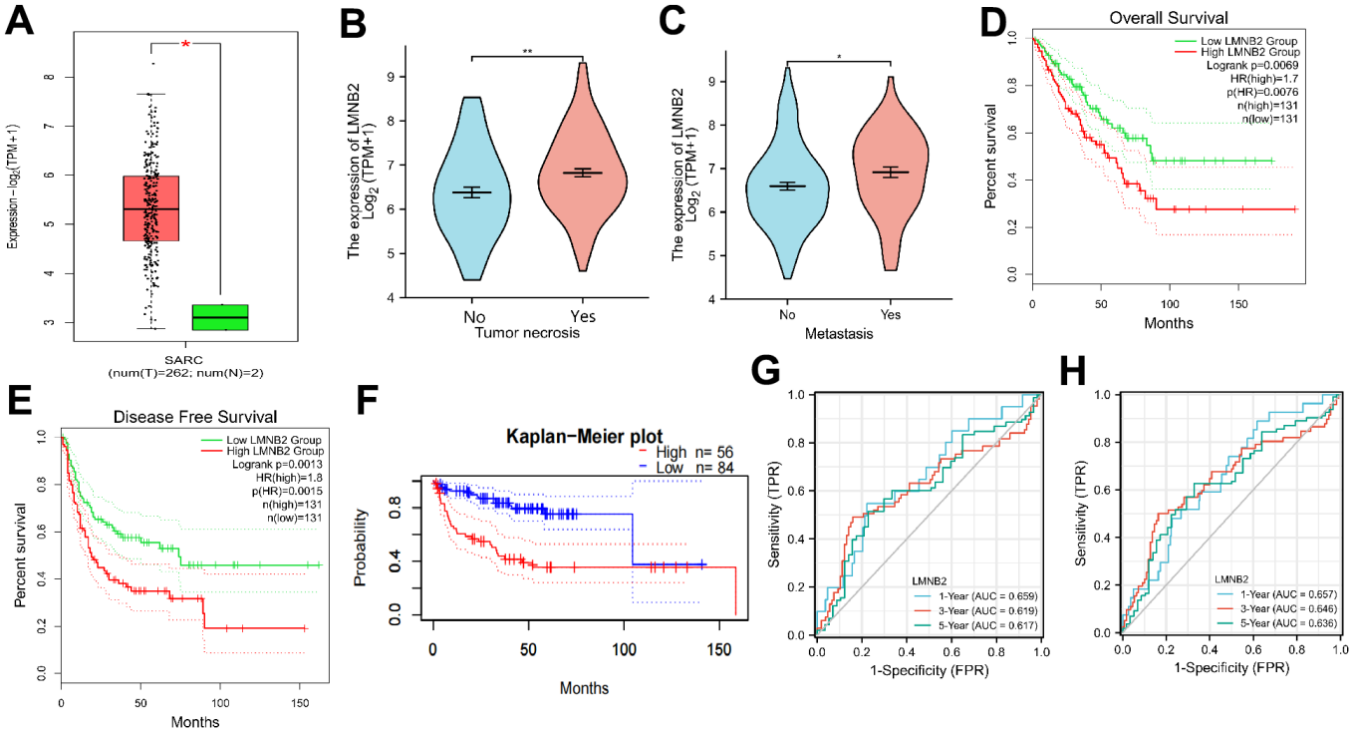
**Prognostic relevance of LMNB2 in SARC.** (**A**) Expression levels of LMNB2 in SARC tissues and normal tissues. Red: SARC tissues; Green: normal tissues. (**B**) Correlation of tumor necrosis and LMNB2 expression level. (**C**) Correlation of tumor metastasis and LMNB2 expression level. (**D**) Kaplan–Meier OS analysis of SARC patients with high or low LMNB2 expression, data from TCGA. (**E**) Kaplan–Meier DFS analysis of SARC patients with high or low LMNB2 expression, data from TCGA. (**F**) Kaplan–Meier DFS analysis of SARC patients with high or low LMNB2 expression, data from GSE30929. (**G**) The time dependent ROC analysis of LMNB2 in predicting SARC patients OS rate was showed. (**H**) The time dependent ROC analysis of LMNB2 in predicting SARC patients DFS rate was demonstrated.

We then used the GEPIA database to analyze the expression level of LMNB2 in SARC. As indicated in Figure 3A, the LMNB2 expression levels in SARC tissues was elevated, when compared with normal tissues. We also analyzed the association between clinico-pathological characteristics and LMNB2 expression levels. A high level of LMNB2 expression showed positively correlation with tumor necrosis (Figure 3B) and metastasis (Figure 3C).

### The prognostic power of LMNB2 in SARC

Univariate and multivariate cox regression were used to detect a possible relation between LMNB2 expression and different clinical factors. The univariate cox analysis revealed that LMNB2 expression(P<0.001), tumor metastasis (P<0.001) and tumor multifocality (P<0.001) showed significant association with the OS rate in SARC (Supplementary Figure 4A). In the multivariate cox regression analysis, LMNB2 expression(P=0.001), tumor metastasis (P<0.001) and tumor multifocality (P=0.014) showed significant correlation with the OS rate (Supplementary Figure 4B). This suggests that LMNB2 might be an independent prognostic factor of SARC. We then asked whether the expression level of LMNB2 affected SARC patients’ prognosis. The median LMNB2value was chosen as cut-off to split SARC patients into a high- or low-expressing group. It was found that elevated LMNB2 expression associated with a worse OS rate and disease-free survival (DFS) rate (Figure 3D, 3E). We continued validating the relation between LMNB2 expression levels and the OS rate by using the GEO database. The results from the GSE30929 cohort also revealed that LMNB2 over-expression was connected with a poorer DFS rate (Figure 3F). The time dependent ROC analysis was carried out to compare the predictive accuracy and risk score of LMNB2 for OS and DFS in SARC. In the OS analysis, the LMNB2 expression level could be used to make a prediction on the prognosis of SARC patients at 1, 3 and 5 years. The ROC’s area under the curve (AUC) was 0.659, 0.619 and 0.617, respectively (Figure 3G) and for the DFS analysis, it was 0.657, 0.646 and 0.636, respectively (Figure 3H).

Altogether, the data suggests that a high expression level of LMNB2 is an indicator for poor prognosis in SARC.

### LMNB2 expression in SARC and its relation with the immune landscape

**Figure 4:**
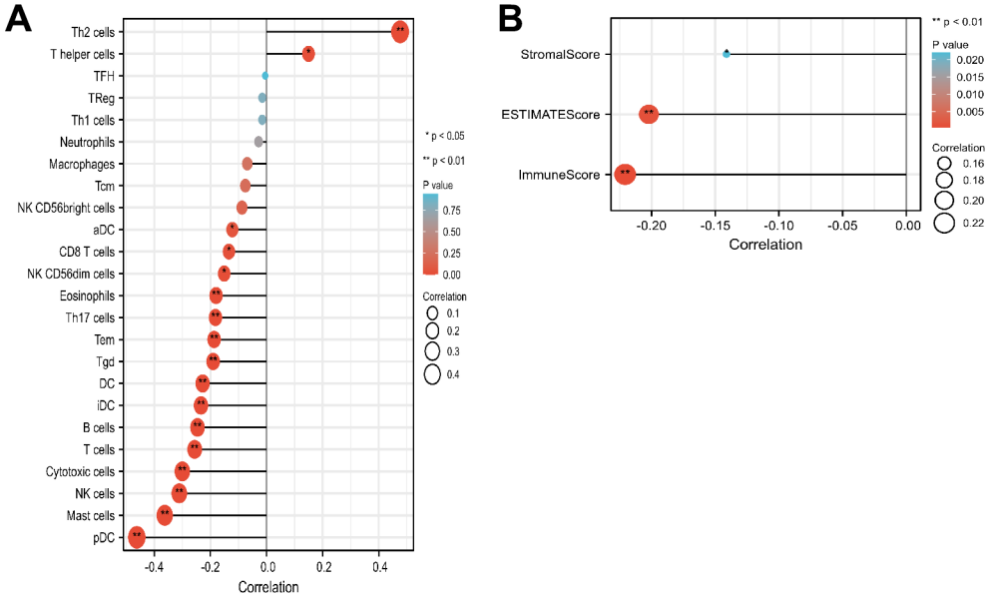
**Correlation of LMNB2 expression with immune landscape in SARC.** (**A**) Correlation analysis of the LMNB2 gene transcript and immune infiltration in SARC. (**B**) Correlation analysis of the LMNB2 gene transcript and estimate score, immune score, and stromal score in SARC.

The relation between LMNB2 and the immune infiltration level in SARC was explored. The expression level of LMNB2 was negatively associated with aDC, B cells, NK CD56 dim cells, CD8 T cells, cytotoxic cells, DC, pDC, Eosinophils, iDC, Mast cells, NK cells, T cells, Tem, Tgd and Th17 cell infiltration. However, LMNB2 expression levels were positively associated withTh2 cells and T helper cells infiltration (Figure 4A). Calculations done with the ESTIMATE algorithm revealed that the expression level of LMNB2 was negatively associated with the immune score, the stromal score, and the estimate score (Figure 4B).

### Co-expression of LMNB2 related genes in SARC

**Figure 5:**
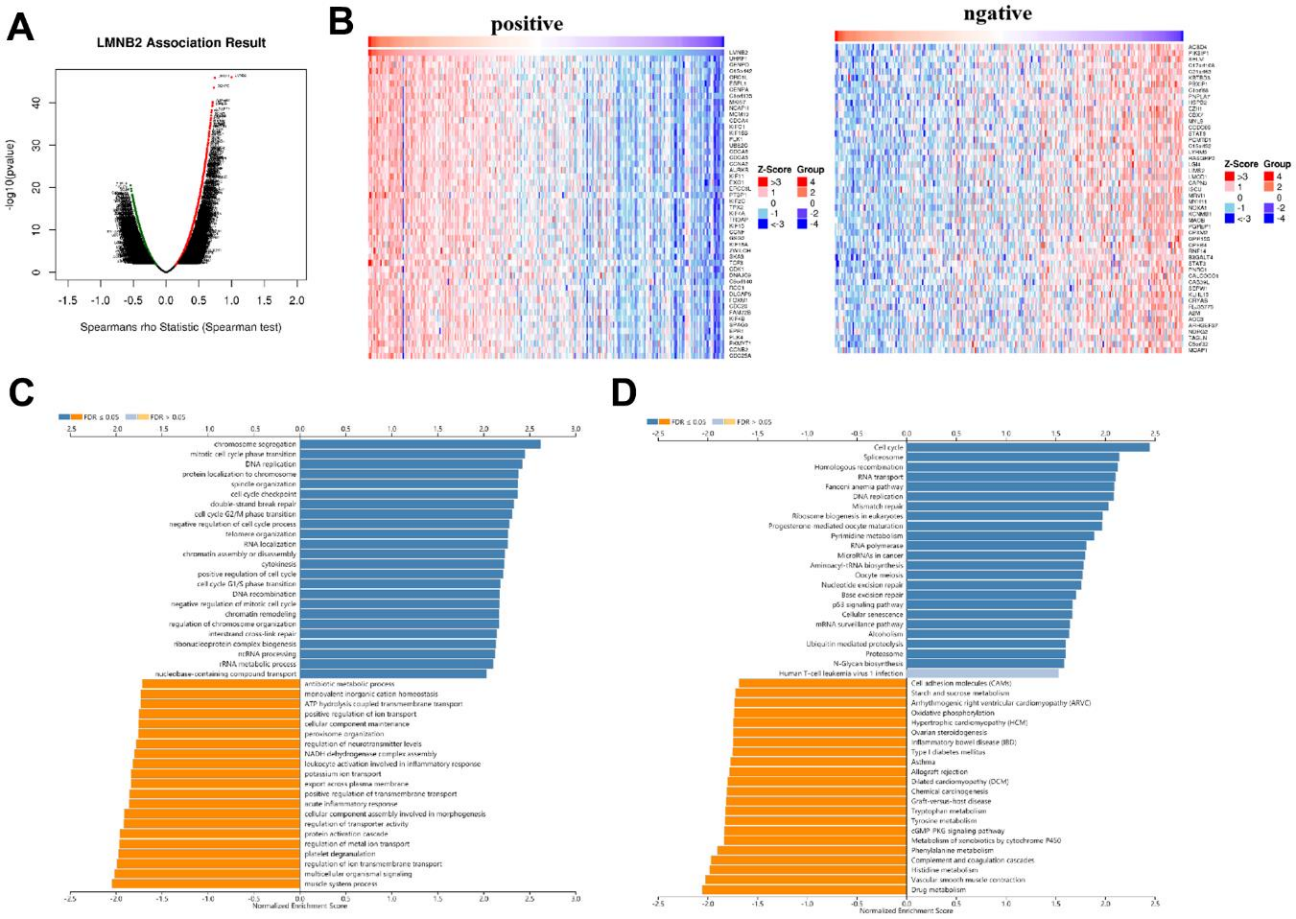
**Co-expression of LMNB2related genes in SARC.** (**A**) The different expression levels of genes are displayed in a volcanic plot. (**B**) Left panel: Top 50 genes that showed positive correlation with LMNB2 expression level. Right panel: Top 50 genes that showed negative correlation with LMNB2 expression level. (**C**) GO_BP of LMNB2 correlated genes in SARC. (**D**) KEGG enrichment analysis of LMNB2 correlated genes in SARC.

LMNB2’s co-expression network in the SARC cohort was analyzed using the Linked Omics database. In Figure 5A, 2843dark red dots represent the genes that show significant positive correlation with LMNB2, while 2756 dark green dots represent the genes that show significant negative correlation with LMNB2 (false discovery rate, FDR < 0.01). 50 genes that showed either positive or negative correlation with LMNB2 expression were selected and displayed in a heat map (Figure 5B). GO annotation of the genes co-expressed with LMNB2 showed that they were mostly involved in mitotic cell cycle phase transition, chromosome segregation, DNA replication, cell cycle checkpoint and other processes (Figure 5C). KEGG database pathway analysis demonstrated that they were primarily involved in DNA replication, the cell cycle, spliceosome, homologous recombination and other pathways (Figure 5D).

### LMNB2 expression status in single cell and its relationship with different cancer functional states

The CancerSEA database (http://biocc.hrbmu.edu.cn/CancerSEA/) was used to look at the expression of LMNB2 in single cells and to explore which functional states were associated with it in different cancers. We found that LMNB2 expression showed a significant positive association with the cell cycle, differentiation, invasion, metastasis and proliferation in most types of cancers (Supplementary Figure 5A). In addition, by mining GSE152048, we further identified the distribution of LMNB2 in tumour microenvironment of SARC (Supplementary Figure 5B).

### LMNB2 down-regulation inhibits cell proliferation by affecting cell cycle pathways

**Figure 6:**
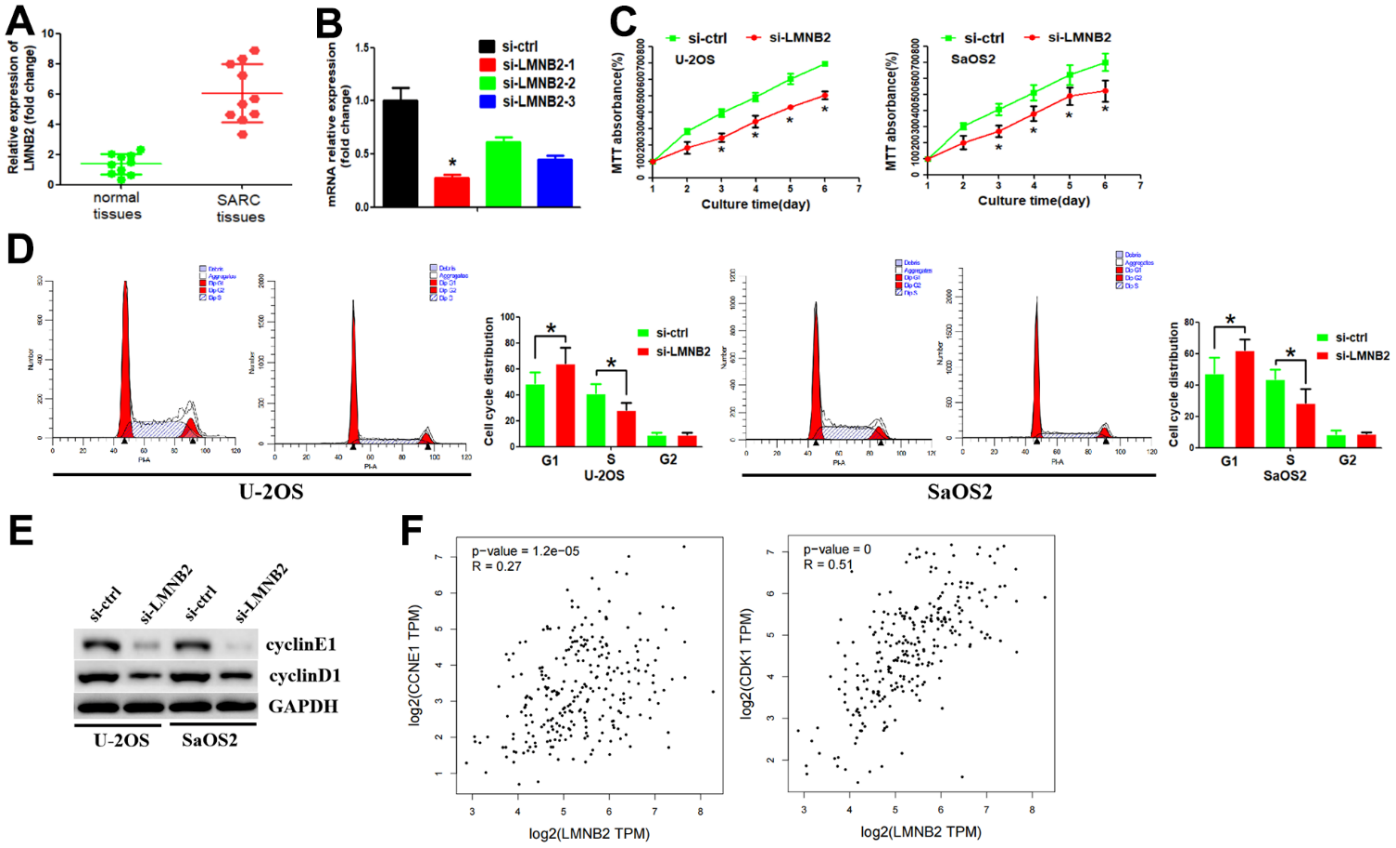
**Down-regulation of LMNB2 inhibited cell proliferation by affecting cell cycle pathways.** (**A**) RT-PCR assay was used to analyze LMNB2 expression levels in SARC tissues and normal tissues. (**B**) The inhibition efficiency of the two si-LMNB2 inhibitors was analyzed by RT-PCR. (**C**) MTT assay was performed to analyze cell proliferation. (**D**) Flow cytometry analysis was performed to analyze the cell cycle distribution. (**E**) Western blot assay was used to determine protein expression levels. (**F**) The association between LMNB2 expression levels and cyclinE1/cyclinD1 expression levels were explored by using the GEPIA2 database.

An RT-PCR assay demonstrated that the expression level of LMNB2 was elevated in SARC tissues when compared with adjacent tissues (Figure 6A). We then used the CCLE dataset to analyze LMNB2 mRNA expression in different SARC cell lines. The result revealed that LMNB2 mRNA was broadly expressed in these cell lines (Supplementary Figure 6).

We examined whether LMNB2 affected cell growth *in vitro*. Therefore, we designed two siRNAs, si-LMNB2-1 and si-LMNB2-2 according the LMNB2 gene. The siRNAs were introduced into the U-2OS and SaOS2 cell line by transfection. The inhibition efficiency was detected by RT-PCR. si-LMNB2-1 displayed the best inhibition efficiency (Figure 6B) and was thus chosen for follow-up study. The MTT assay showed that down-regulation of LMNB2 significantly decreased cell proliferation (Figure 6C). Because KEGG enrichment and GO analysis showed that LMNB2 may be involved in the cell cycle regulation, we asked whether LMNB2 regulated cell proliferation by affecting the cell cycle distribution. Flow cytometry revealed that in si-LMNB2-1 transfected cells the number of cells in the G1 phase was increased and in the S phase decreased (Figure 6D). This finding suggests that LMNB2 regulates the G1/S cell cycle checkpoint. We therefore analyzed the effect of LMNB2 down-regulation on the regulators of cell cycle progression at the G1/S checkpoint. LMNB2 down-regulation decreased cyclinD1 and cyclinE1 levels (Figure 6E). In parallel, we revealed that there was a positive association between LMNB2 and cyclinD1 and cyclinE1 (Figure 6F). Furthermore, cyclinB1, which is involved in the G2/M phase was not affected (data not shown).

### LMNB2’s affection on cancer stem cell properties and chemo-sensitivity

**Figure 7:**
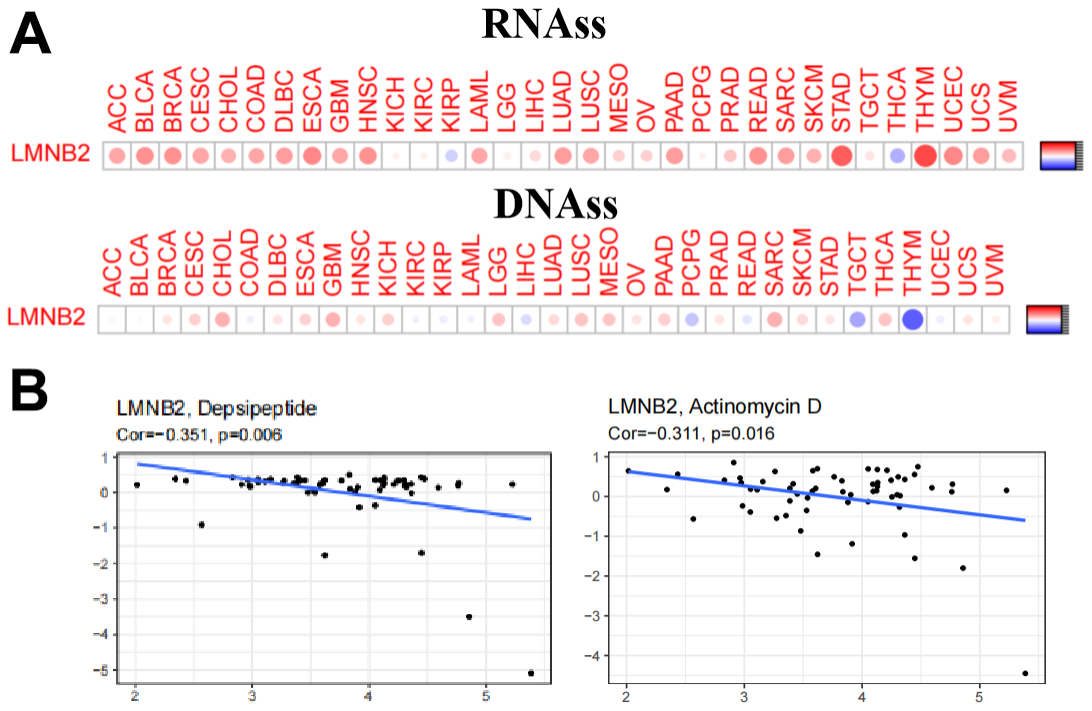
**LMNB2’s affection on cancer stem cell properties and chemo-sensitivity.** (**A**) The correlation between the LMNB2 gene and tumor stem cells was measured by the DNA stem cell score (DNAss) and RNA stem cell score (RNAss) based on the DNA methylation pattern and mRNA expression respectively. (**B**) LMNB2 expression level was associated with cellular resistance to depsipeptide and actinomycin D for the treatment of SARC.

During cancer progression, tumor cells can over time lose their differentiation phenotype thereby obtaining progenitor and stem cell like characteristics. The correlation between the LMNB2 gene and tumor stem cells was measured by the DNA stem cell score (DNAss) and RNA stem cell score (RNAss) based on the DNA methylation pattern and mRNA expression respectively (Figure 7A). LMNB2 showed varying degrees of association with RNAs and DNAs in different cancer types and the expression level of LMNB2 showed positive correlation with RNAss and DNAss in SARC. This suggests that LMNB2 might be involved in stem cell like characteristics.

Next, we studied the expression of the LMNB2 gene in the NCI-60 cancer cell line panel and systematically tested the correlation between its expression level and the sensitivity of more than 200 chemotherapeutic drugs. Interestingly, we found that an increased expression of LMNB2 was related to an increased resistance of different cell lines to a variety of chemotherapeutic drugs (r > 0.3, P < 0.005). Among these drugs, we noted that LMNB2 was associated with cellular resistance to depsipeptide and actinomycin D for the treatment of SARC (Figure 7B).

## DISCUSSION

Despite the variety of cancer treatments, the prognosis of multiple types of cancer is still not ideal. There is an urgent need to identify tumor-associated genes to better understand the occurrence, maintenance and progression of cancer. Laminin proteins are overexpressed in a lot of cancers and have an ability to maintain homeostasis in cancer cells [8, 9]. For example, Lamin B binding may have a differential influence on gene expression by forming complexes with different epigenetic regulators, transcription factors and chromatin binding proteins in specific chromatin regions [10, 11]. It is known that LMNB2 can also play an important role in tumor development [12]. However, the relation between LMNB2 expression and cancer progression is still poorly understood.

We first used GTEx and TCGA datasets to evaluate the expression and prognostic significance of LMNB2 in generalized carcinoma. Our data showed that LMNB2 mRNA has a high expression in almost all types of cancer, with the exception of PAAD and PCPG, when compared with both adjacent and normal tissues. This result suggests that LMNB2 may be an oncogene. Kaplan-Meier and univariate Cox regression analysis showed that up-regulated expression of LMNB2 correlated with adverse OS effects in ACC, KIRP, LGG, LIHC, LUAD, MESO, PRAD, SARC, and SKCM. However, in STAD and THYM, high expression of LMNB2 was associated with better OS, suggesting that LMNB2 has a more protective role in both cancers. In addition, we found that up-regulation of LMNB2 expression was associated with shortened PFS in ACC, BLCA, BRCA, LGG, MESO, PAAD, PRAD, and SARC patients. These findings indicate that LMNB2 could be possible biomarker for prognosis prediction of cancer patients.

Earlier studies showed that the immune status of a tumor is closely related to the cell composition and invasion in the tumor environment [13]. We showed that LMNB2 can play a crucial role in cancer immunity. According to the mRNA expression profile, the ESTIMATE algorithm can calculate the following scores to evaluate the abundance of immune invasion; the immune score that denotes the infiltration of immune cells in tumor tissue, the stromal score that captures the presence of stroma in tumor tissue, and the estimate score that infers tumor purity. We showed that LMNB2 was negatively correlated with all three.

The TMB shows the overall neoantigen load within the tumor and is therefore directly linked to immunotherapy efficiency [14, 15]. MSI is also a key biomarker of the immune checkpoint inhibitors (ICI) response [16, 17]. Earlier, the US Food and Drug Administration (FDA) approved high microsatellite instability (MSI-H) and defect mismatch repair (dMMR) as prognostic biomarkers to guide the clinical use of ICIs in some cancers. In this study, we demonstrated that LMNB2 expression is associated with TMB and MSI in several cancer types. In conclusion, LMNB2 may be a potential predictor of immunotherapy efficacy for these types of cancer.

We then focused on LMNB2’s function in SARC. In our analysis, we revealed that LMNB2 expression levels were up-regulated in SARC tissues and cell lines. The biological function assays revealed that LMNB2 down-regulation inhibited cell proliferation by affecting the cell cycle distribution. LMNB2 affected cylinD1 and cyclinE1 expression and thereby contributed to cell cycle arrest in the G1 phase. However, further investigation is needed to discover how LMNB2 regulated cylinD1 and cyclinE1 expression.

In conclusion, our study shows that the expression profile of LMNB2 has significant prognostic value in pan-cancer, especially SARC. LMNB2 expression levels correlated with TME, TMB, MSI, the stemness score and immune subtype. In addition, comprehensive analysis found that LMNB2 was a new oncogene in SARC, which was confirmed *in vitro*. These results may provide new insights for future studies on the potential targets of LMNB2 in pan-cancer.

## MATERIALS AND METHODS

### Data collection and analysis

The TCGA database was used to examine the gene expression RNA-seq dataset and clinical records of 11058 patients (http://xena.ucsc.edu/welcome-to-ucsc-xena/, workflow type: HTSeq FPKM).

The effects of LMNB2 gene expression and clinical characteristics for the prognosis of SARC were determined. The R package “forestplot” was used to generate a forest plot of the univariate and multivariate Cox regression analysis. The Kaplan-Meier survival curve was analyzed with the R packages “Survival” and “Survminer”. Overall survival (OS) and progression-free survival (PFS) rates were calculated by the Mantel-Cox test.

The tumor cell line mRNA expression matrix was derived from the CCLE data set (https://portals.broadinstitute.org/ccle) andanalyzed by R package “ggplot2”. The predictive accuracy and risk score of LMNB2 were compared by ROC analysis. R software version V4.0.3 was used.

The co-expression network of LMNB2 in the SARC cohort was analyzed and visualized in the LinkedOmicsportal (http://www.linkedomics.org/login.php).

### Tumor microenvironment, microsatellite instability, tumor mutational burden and stemness analysis

The stromalscore, estimate score and immune score were calculated using the R packages”Estimate” and “Limma”. The stemness scores for DNA (DNAss) and RNA (RNAss) were taken from Xena (https://xenabrowser.net/datapages/, version 07-20-2019). The Spearman correlation method and R package “Corrplot” were used to evaluate the RNAss and DNAss.

### Cell lines culture and si-RNA transfection

SARC cell lines U-2OS and SaOS2 were maintained in PRMI-1640 medium mixed with 10% fetal bovine serum and 1% penicillin-streptomycin at 37° C with 5% CO_2_.

The target sequences of LMNB2 si-RNA-1 were 5′-CCTCGGTGATGCGTGAGAATGAGAA-3′ and for si-RNA-2 5′-GAGGTCAACAAGAGCGCCAAGAAGA-3′. Transfection was performed according manufacturer’s instructions. The details were as follows: 24 hours before transfection, cells were seeded onto a 6-well plate at 40–50% confluence. The siRNAs were then transfected at a working concentration of 100 nM using TurboFect siRNA Transfection Reagent. After 48–72 hours, cells were collected and used for subsequent experiments.

### SARC tissues collection and RNA extraction

For RNA extraction, RNA was extracted from cells by using Trizol and cDNA was generated by a reverse transcription reagent kit from TaKaRa company. Subsequently, the cDNA template was used for amplification with specific primers. Primers for LMNB2 PCR were: forward primer5′-GTCCTGGATGAGACGGCTC-3′, reverse primer5′-GCGCTCTTGTTGACCTCGT-3′. Primers for GAPDH PCR were: forward primer5′-GGAGCGAGATCCCTCCAAAAT-3′, reverse primer5′-GGCTGTTGTCATACTTCTCATGG-3′.

### MTT cell viability assay

The MTT Cell Viability Assay kit was used as follows; cells were added to a 96-well plate and 5 mg/mL MTT was added. After a 4h incubation the precipitation was dissolved in DMSO and the absorbance was measured with a microplate spectrophotometer at 490 nm.

### Cell cycle analysis

For cell cycle analysis, cells after incubation for 48 h were harvested, followed by washed with cold PBS. Then the cells were fixed with 70% ice-cold ethanol at 4° C overnight. Subsequently, the cells were incubated with PBS, which contained propidium iodide and RNase A. Finally, the fixed cells were washed with cold PBS three times. FACS caliber flow cytometry (BD Biosciences, USA) was used to gain the DNA content of labeled cells.

### Western blot assay

Total protein from cells was extracted with protease inhibitor containing RIPA lysis buffer. The BCA protein method was used to determine the protein concentration. Subsequently, proteins were separated by 10% SDS-PAGE gel electrophoresis and transferred onto PVDF membranes and blocked for 1 hr at room temperature in 5% skim milk. Then, all membranes were incubated with primary antibodies: Anti lmnb2, anti cyclin1, anti cycline1 and anti GAPDH at 4° C overnight. After that the membranes were washed with TBST three times, incubated with secondary antibody at room temperature for 1.5 hr, washed four times with TBL reagent and analyzed. The immunoblots were detected with an imaging system (Bio-Rad, USA) by using enhanced chemiluminescence detection kit. The concentration of primary antibodies used were listed as follows: LMNB2,1:500 (Abcam, ab151735); cyclinE1, 1:500(Abcam, ab133266); cyclinD1, (Abcam, ab16663) and GAPDH,1:500 (Abcam, ab8245).

### Availability of date and materials

The data that support the findings of this study are available from the corresponding author, upon reasonable request.

## Supplementary Material

Supplementary Figures
