# Supplementary material for: Comprehensive analysis of LMNB2 in pan-cancer and identification of its biological role in sarcoma
Source: Aging (Albany NY). 2024 Jul 8;17(1):203–16. doi: 10.18632/aging.205962 (PMC11810055; doi:10.18632/aging.205962)
Supplement: Supplementary Figures [file aging-17-205962-s001.pdf]

SUPPLEMENTARY FIGURES

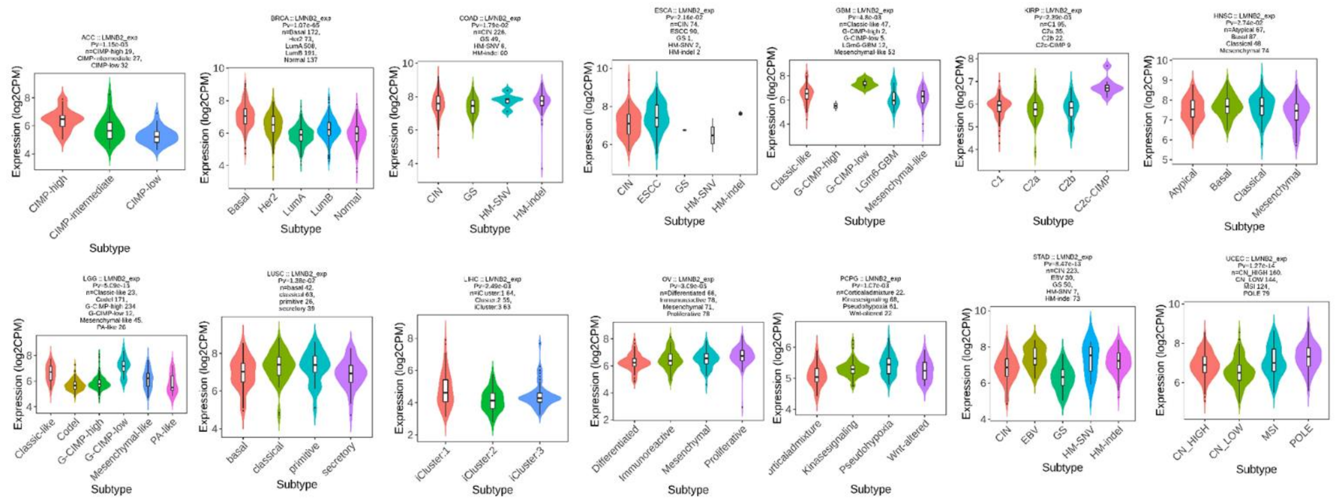

Supplementary Figure 1. LMNB2 expression levels in different molecular subtypes of cancer are indicated.

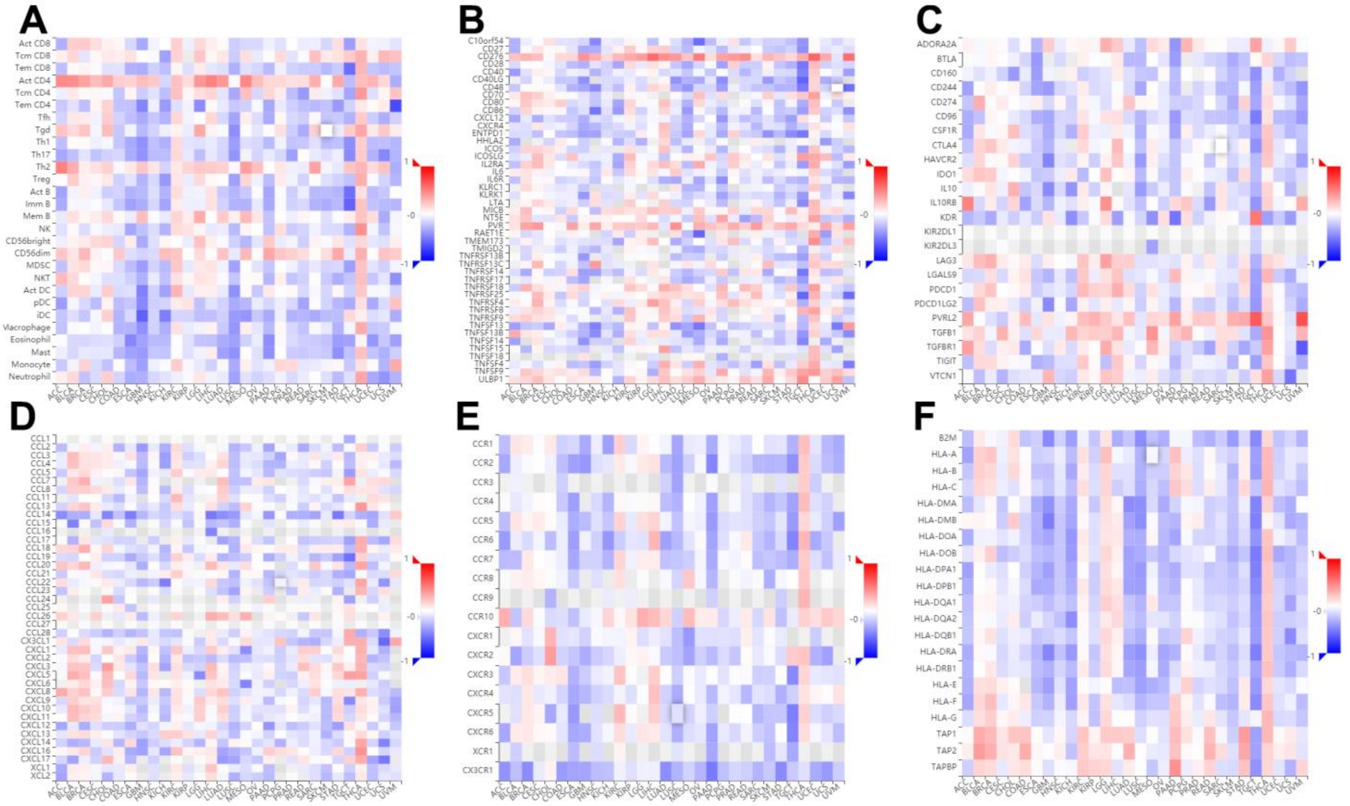

Supplementary Figure 2. Relation between the (**A**) abundance of tumor-infiltrating lymphocytes; (**B**) immunostimulator; (**C**) Immunoinhibitor; (**D**) chemokines; (**E**) chemokine receptors and (**F**) MHC molecule and expression of LMNB2.

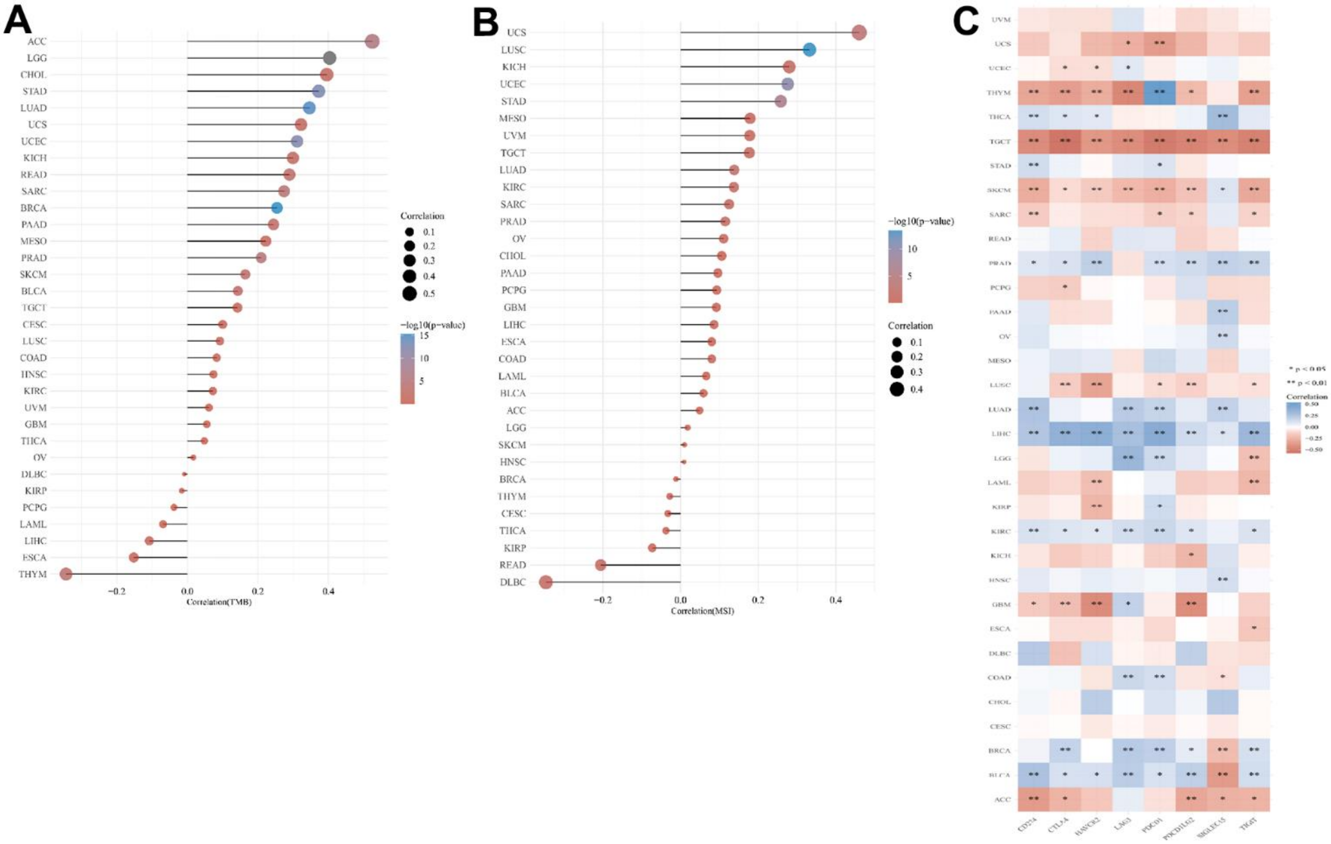

Supplementary Figure 3. Spearman correlation analysis of (**A**) TMB; (**B**) MSI and (**C**) immune markers and LMNB2 gene expression.

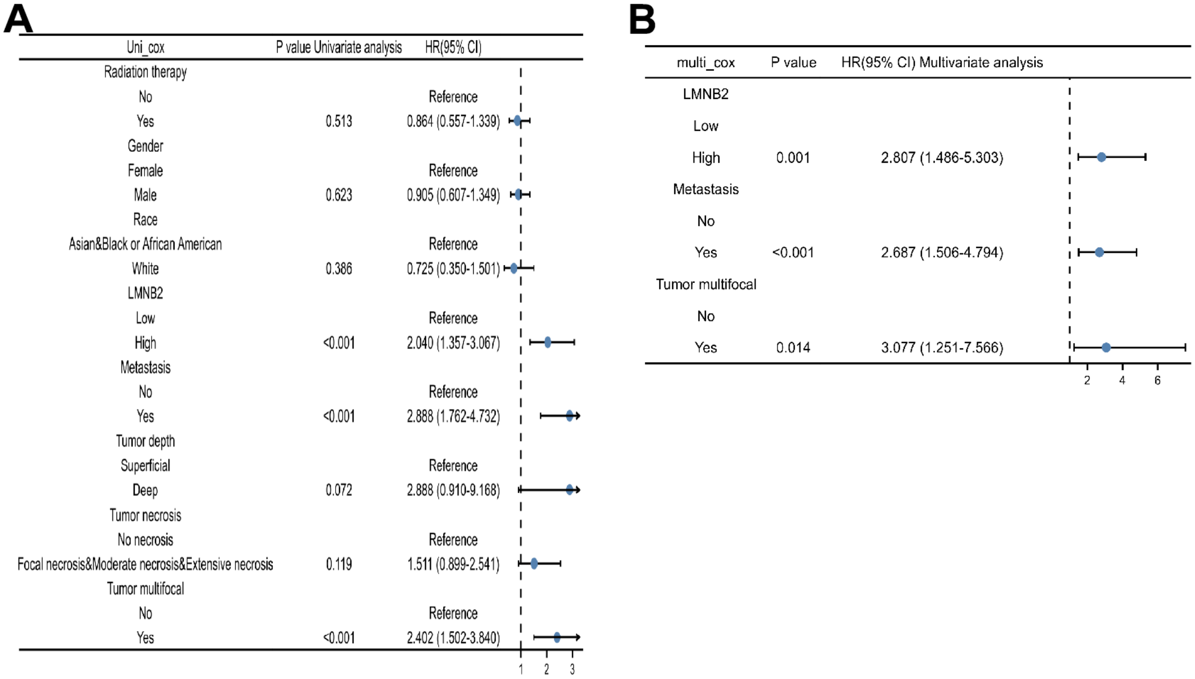

Supplementary Figure 4. Forest plot showing the (**A**) univariate Cox regression and (**B**) multivariate Cox regression analysis results in SARC patients.

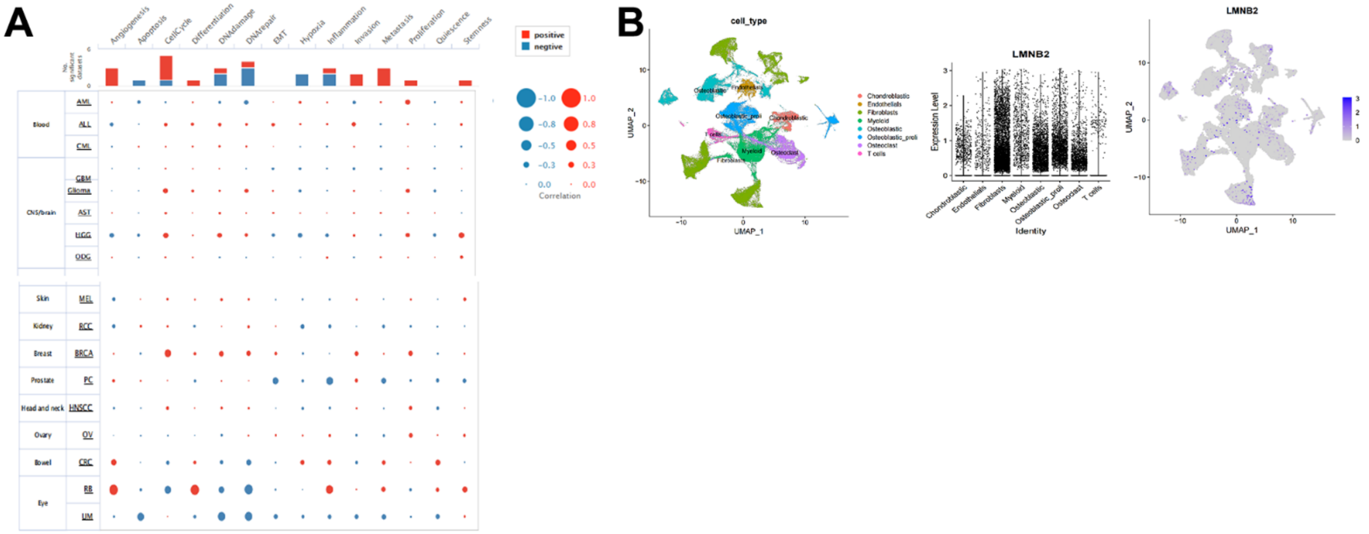

Supplementary Figure 5. (**A**) The expression levels of LMNB2 and its relationship with tumor functional status across different cancers in single cell, data from CancerSEA database. (**B**) The distribution of LMNB2 in TME of SARC tissues, data from GSE152048.

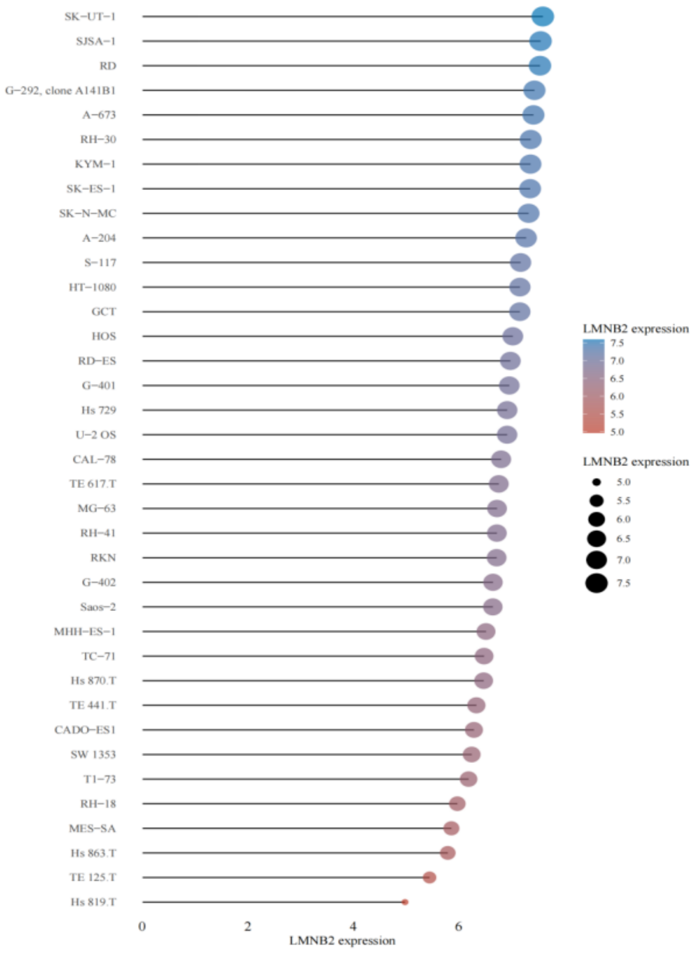

Supplementary Figure 6. LMNB2 mRNA expression levels in different SARC cell lines were analyzed, data from CCLE dataset.
